# Innovation focus in 2023

**DOI:** 10.1016/j.xinn.2023.100560

**Published:** 2024-01-02

**Authors:** The Innovation Editorial Team, China

Review of scientific breakthroughs in 2023, from neuromodulation and brain-machine interfaces to AI, superconductivity, and space exploration by The Innovation’s editorial team (Figure 1).

**Figure 1:**
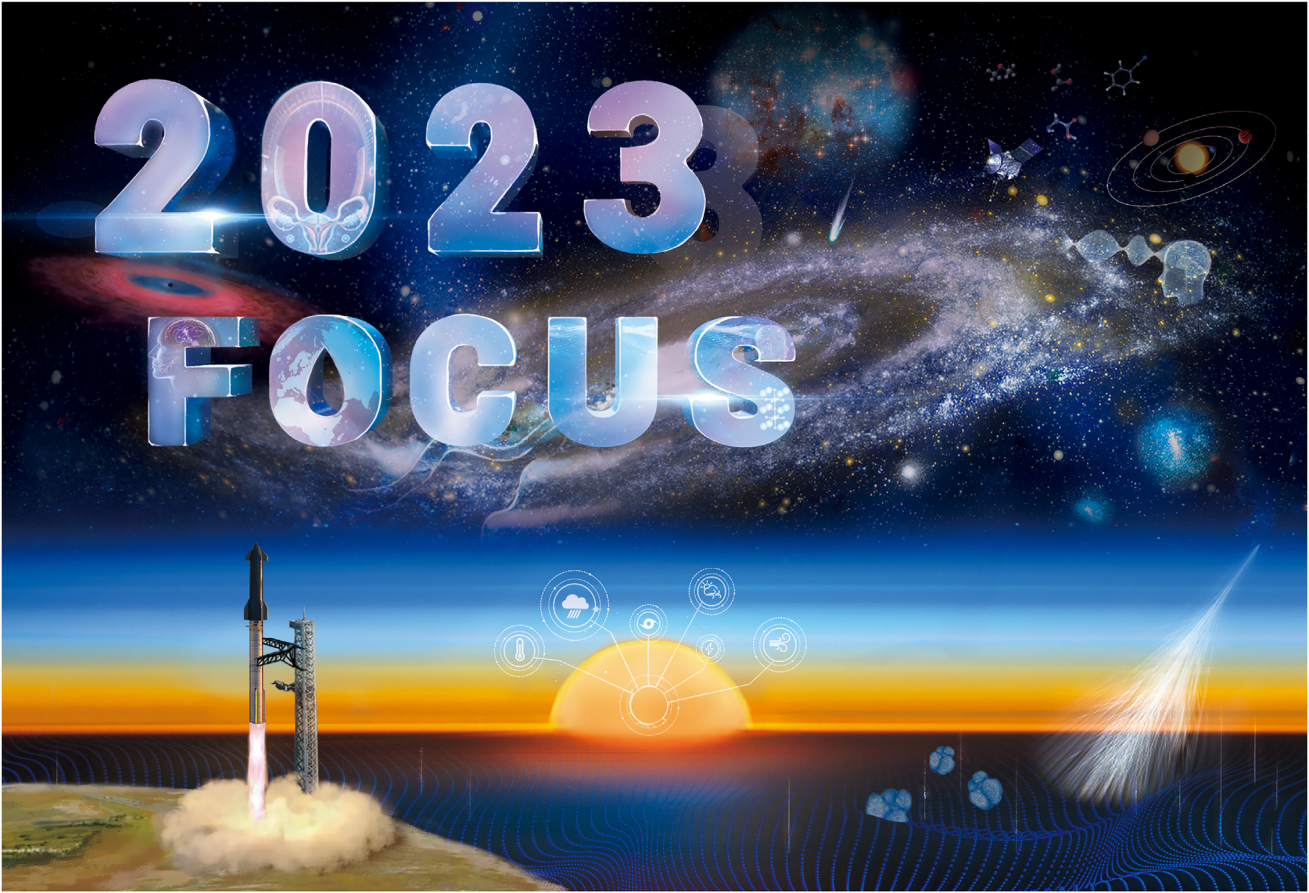
Review of scientific breakthroughs in 2023, from neuromodulation and brain-machine interfaces to AI, superconductivity, and space exploration by *The Innovation*’s editorial team

## Neuromodulation leads to breakthroughs

Neuromodulation aims to modulate the activity of specific neurons through physical, chemical, or biological means, thereby modifying related perception, action, or even reversing disease states. Among the currently available neuromodulation techniques, chemogenetics has emerged as a rising star in recent years, offering advantages such as cell-type and pathway specificity, less invasiveness, and increased flexibility. Through the use of this promising technique, researchers worldwide have achieved significant breakthroughs in 2023. One notable example is the world’s first cell-level hypothermia experiment on primates conducted by Drs. Dai and Wang, which successfully induced a drop in body temperature and triggered a strong defensive response against the cold by activating specific neurons in the monkeys’ brains. This represents a significant advancement in understanding human torpor and its potential applications in medicine and space exploration. Another breakthrough is the development of a gene therapy strategy by Dr. Lu and collaborators aimed at selectively manipulating the circuitry affected by Parkinson’s disease and attenuating its core motor symptoms in rodent and non-human primate animals. This strategy brings new hope for the treatment of Parkinson’s disease.

## Functional ultrasound brain-machine interface

Non-implanted brain-machine interfaces (BMIs) have the potential for various applications such as biomedicine, consumer electronics, and smart manufacturing. A team of scientists has made important progress in decoding motor plans using a non-implanted and closed-loop online functional ultrasound BMI (fUS-BMI). They used a miniaturized 15.6 MHz ultrasound transducer to record the neural activities during the ultrafast pulse-echo fUS neuroimaging. The experiments performed on the posterior parietal cortex of two trained monkeys achieved real-time control of up to 8 motion directions and stable decoding for over a month. The technological breakthrough established the feasibility of fUS-BMI and paved a way for a less-invasive and portable human BMI, which is high resolution, stable across time, and scalable to sense activities from larger and deeper brain regions. To make fUS-BMI for humans a reality in the future, several cutting-edge technological innovations should be embarked upon, especially new techniques to overcome ultrasound signal attenuation in non-craniotomy mode.

## OvaRePred: Redefining ovarian aging

The ovarian reserve, indicative of the number of primordial follicles in the ovarian cortex that can mature into oocytes, is a vital fertility marker in women. This reserve varies dramatically, spanning from thousands to millions at birth. Traditionally, ovarian reserve assessment relies on clinicians’ personal experiences. OvaRePred, utilizing advanced algorithms and big data, offers insights into current ovarian health and predicts key fertility milestones, such as the onset age of diminished ovarian reserve and perimenopause. Its capability to evaluate and forecast ovarian reserve status paves the way for individualized care, informed decision-making, and an enhanced quality of life. OvaRePred is currently being routinely implemented in several hospitals and physical examination centers in China. As its application becomes more widespread, it promises to deepen our understanding of fertility and perimenopause, culminating in improved health management for individuals globally. This tool represents not just a significant milestone in medical technology; it also signals the onset of a revolutionary era in precision reproductive health management.

## Beyond LLM, AI is moving to multimodal foundation models

Just 1 month after the emergence of ChatGPT, Innovation focus 2022 accurately predicted the profound impact that large models would have on the development of human technology. Indeed, these artificial intelligence (AI) technologies have made their way into the progress lists of *Nature* and *Science* this year. In 2023, large models have transcended the confines of natural language processing and have successively emerged as foundation models for multimodal data and various AI tasks. Huawei’s Pangu-Weather and Google DeepMind’s GraphCast utilized AI foundation model techniques and achieved a breakthrough in more accurate weather forecasting. OpenAI’s GPT-4 Turbo showcased outstanding capabilities in processing images, text, and speech. Google’s Gemini achieved results that surpass human capabilities in multiple tasks. The continuous advancement of large foundation models is pushing the boundaries of AI capabilities and gradually transforming the way people interact with the world.

## Analyzing samples from asteroids Ryugu and Bennu

Organic compounds in exogenous materials are thought to have significantly contributed to prebiotic chemistry on Earth. Japan’s Hayabusa 2 mission and the United States’ OSIRIS-Rex mission collected samples from the carbonaceous asteroids Ryugu and Bennu and delivered them to Earth. Analyzing these samples in the laboratory revealed approximately 20,000 organic molecular species with molecular weights of up to 700 Da in the Ryugu sample, including indigenous amino acids, nucleobase, vitamer, monocarboxylic acids, alkylamines, and polycyclic aromatic hydrocarbons. The Ryugu sample’s organic species distribution is as diverse as that in carbonaceous meteorites, probably representing the real distribution of organics in the solar system. The analysis of the Bennu sample also shows high carbon and water content. These findings will help advance our understanding of the early history of our solar system and the origin of life.

## Deciphering the most violent dynamics of the universe

The observation of gamma-ray bursts (GRBs) and extremely energetic cosmic rays can offer valuable insights into the formation and evolution of stars and galaxies. The Large High Altitude Air Shower Observatory (LHAASO) achieved a groundbreaking observation of tera-electron volt afterglow onset and decay phases of an exceptionally bright GRB, 221009A. GRBs with such brightness are extremely rare, being expected to occur only once every ten thousand years. This observation led to the finding of a jet break of the GRB that explains its brightness nature and the first-ever discovery of a rapid rise to a peak in the afterglow’s light curve, which is still a mystery. Another notable discovery reported this year is that the Telescope Array experiment’s surface detector array detected a cosmic ray with an energy level of around 200 exa-electron volts on May 27, 2021. Scientists are actively researching the origins of these energetic cosmic rays and the mechanisms responsible for accelerating them to such extraordinary energies. Overall, both the observations of GRBs and extremely energetic cosmic rays provide valuable data to expand our understanding of the universe.

## Decoding the Y chromosome

When the preliminary human genome sequence was assembled over two decades ago, gaps riddled the maps of all chromosomes. However, unlike tiny scattered gaps in other chromosomes, over half of the Y chromosome’s sequence remained a mystery, with its highly repetitive DNA impervious to decoding. For the last 22 years, scientists have struggled to interpret the Y chromosome’s genetic information. To tackle the most repetitive regions of the genome, the T2T Consortium leveraged new DNA sequencing approaches, sequence assembly methodology, and knowledge from generating the first complete sequences of the other 23 human chromosomes. In August, researchers at NIST and partner institutions integrated a comprehensive picture of the Y chromosome, uncovering 42 novel genes and establishing a solid basis for analyzing chromosomal structure and function. Sequencing revealed unexpected uniformity within the Y chromosome, with nearly half composed of alternating blocks of two specific repeating satellite DNA sequences in an orderly, quilt-like pattern. Additionally, deciphering repetitive regions involved in sperm production and containing variable gene copies allows for a more accurate assessment of DNA deletions and connections between genomic variation, fertility, disease, and health.

## Recent decline in global water availability

Water availability across the globe plays a crucial role in shaping livelihoods, socioeconomic development, and ecosystems. However, it is anticipated that this availability will undergo substantial transformations in the next few decades due to the combined effects of climate change and socioeconomic growth. A group of scientists found that the Southern Hemisphere was responsible for the declining trend in global water availability in the early 21st century (from 2001 to 2020), with significant decreases occurring specifically in South America, southwestern Africa, and northwestern Australia. In contrast, the Northern Hemisphere displayed complex regional trends that offset each other, leading to a negligible overall trend. The Southern Hemisphere’s water availability trends were predominantly driven by precipitation associated with climate modes, particularly the El Niño-Southern Oscillation, emphasizing their critical role in regulating global water availability.

## Rising nickel age of superconductivity

Superconductivity is a macroscopic quantum phenomenon with zero resistance and full diamagnetism states at low temperatures. Over the past 36 years, the copper oxides have been the only unconventional superconductors that exhibit bulk superconductivity with T_c_ above 77 K, a threshold for practical applications. Since 2019, superconductivity has been discovered in a thin nickelate film NdNiO_2_ with T_c_ up to 15 K. A great breakthrough was made by Sun et al. this year, revealing superconductivity up to 80 K in its cousin compound, La_3_Ni_2_O_7_, at pressures between 14.0 and 43.5 GPa. Transition-metal oxides have been intensively explored for many years to search for unconventional superconductivity. Now, a new chapter of the nickel age of superconductivity has finally opened, not only in the 112-type and 327-type of nickelates but also in many related compounds named Ruddlesden-Popper phases with a formula La_n+1_Ni_n_O_3n+1_ (n = 1, 2, 3, …, ∞). Indeed, they show many aspects of similarity to cuprates, as well as iron-based superconductors, such as competing magnetic and charge orders, sign reversal pairing symmetry, and hybridized orbitals, where strong magnetic fluctuations may play the pairing glue of superconducting electrons.

## What is next for space exploration

SpaceX’s Starship spacecraft and Super Heavy rocket collectively referred to as Starship represent a fully reusable transportation system designed to carry both crew and cargo to Earth orbit, the Moon, Mars, and beyond. SpaceX also has crewed spaceflights with private passengers aboard Starship planned for later this decade. In 2021, Isaacman flew on Inspiration-4, SpaceX’s first all-civilian Crew Dragon flight, and will also lead the first Polaris flight aboard a SpaceX Dragon early next year. Although a second test flight of SpaceX's Starship was failed on November 12, 2023, the spacecraft could potentially transport astronauts and private passengers to the moon within the next several years, and possibly to Mars later on. China also conducted three commercial rocket launches in December, 2023. The solid carrier rocket Gui Shen Xing-1, developed by Galaxy Power, successfully completed the first orbital launch mission by a private rocket company, covering the period from dawn to dusk. Additionally, Zhuque-2 is the world's first liquid oxygen-methane carrier rocket to achieve consecutive successful launches, marking China's commercial space exploration entering the fast lane globally. In the future of space exploration, commercialization is an inevitable trend.

